# Impact of body composition on pathological response to neoadjuvant immunotherapy in dMMR/MSI-H colorectal cancer

**DOI:** 10.3389/fimmu.2025.1589869

**Published:** 2025-05-30

**Authors:** Ziyang Zeng, Jian Zhou, Weili Zhang, Jianhong Peng, Yuan Li, E-er-man-bie-ke Jin-si-han, Hao Wang, Shaopu Lian, Cheng Feng, Chuanmiao Xie, Zhizhong Pan, Zhenhai Lu

**Affiliations:** ^1^ Department of Colorectal Surgery, State Key Laboratory of Oncology in South China, Guangdong Provincial Clinical Research Center for Cancer, Sun Yat-sen University Cancer Center, Guangzhou, China; ^2^ State Key Laboratory of Oncology in South China, Guangdong Provincial Clinical Research Center for Cancer, Department of Radiology, Sun Yat-sen University Cancer Center, Guangzhou, China; ^3^ Guangdong Key Laboratory of Nasopharyngeal Carcinoma Diagnosis and Therapy, Guangdong Provincial Clinical Research Center for Cancer, Department of Radiology, Sun Yat-sen University Cancer Center, Guangzhou, China; ^4^ South China Hospital, Medical School, Shenzhen University, Shenzhen, China; ^5^ Department of Thyroid & Galactophore Surgery, People’s Hospital of Longhua, Shenzhen, China; ^6^ State Key Laboratory of Oncology in South China, Guangdong Key Laboratory of Nasopharyngeal Carcinoma Diagnosis and Therapy, Guangdong Provincial Clinical Research Center for Cancer, Department of Radiology, Sun Yat-sen University Cancer Center, Guangzhou, China

**Keywords:** MSI-H, dMMR, colorectal cancer, PD-1 blockade, body composition, obesity

## Abstract

**Background:**

Obesity and overweight have been suggested as a potential predictor of favorable outcomes in certain malignancies treated with immunotherapy. However, most studies have relied on BMI as a proxy for adiposity, without fully considering the distinct roles of fat and lean tissues. This study aimed to explore the association between body composition and treatment response in colorectal cancer patients receiving neoadjuvant PD-1 inhibitor therapy.

**Methods:**

Patients with dMMR/MSI-H colorectal cancer undergoing neoadjuvant PD-1 blockade were included in this study. Body composition parameters were measured using baseline CT images. Pathological response was assessed using tumor regression grade (TRG). Univariate and multivariate analyses were performed to examine the association between body composition variables (total adipose tissue, visceral adipose tissue, subcutaneous adipose tissue, visceral-to-subcutaneous adipose tissue ratio, and skeletal muscle) and pathological complete response (pCR) rates. Correlation analysis was conducted to detect the relationship between body composition and lipid profiles.

**Results:**

A total of 84 patients were included in the analysis. Patients with poor treatment response exhibited significantly lower levels of visceral adipose tissue (VAT), total adipose tissue (TAT), and BMI. On multivariate analysis, higher VAT volume and elevated circulating lymphocyte count were independently associated with increased pCR rates. The positive association between VAT and treatment response was consistent across most subgroups except in patients aged ≥ 65, where the effect tended to be reversed. Additionally, VAT volume correlated positively with triglycerides and negatively with high-density lipoprotein cholesterol.

**Conclusion:**

Higher visceral adipose tissue volume is associated with improved pathological complete response in dMMR/MSI-H colorectal cancer patients treated with PD-1 inhibitors. However, this favorable effect of visceral adiposity may be diminished or reversed in elderly patients (≥ 65 y), highlighting the potential influence of aging on the metabolic-immune interplay in immunotherapy response.

## Introduction

Colorectal cancer (CRC) is the third most common type of cancer and the second leading cause of cancer-related deaths worldwide (1). Recent advances in immunotherapy have revolutionized the treatment landscape of colorectal cancer, particularly for patients with microsatellite instability-high (MSI-H) and mismatch repair deficient (dMMR) tumors (2–4). This type of tumor, which accounts for 12%~15% of total colorectal cancer, is characterized by a high mutation burden, and particularly susceptible to immune checkpoint inhibitors (ICIs) (5). However, not all patients benefit equally, and the objective response rates in metastatic CRC patients receiving PD-1 inhibitors only varied from 31.1% to 52% across different studies (3, 6–9).

Beyond metastatic settings, PD-1 inhibitors have been increasingly utilized in neoadjuvant therapy for non-metastatic, locally advanced CRC, aiming to enhance tumor response rates and potentially reduce the need for invasive surgical interventions. Neoadjuvant immunotherapy has demonstrated remarkable tumor regression in dMMR/MSI-H colorectal cancer, with pathological complete response (pCR) rates reported at 60%, 67%, 69%, 80% and 88% in various studies (10–14). Despite its superior efficacy compared to chemotherapy, predictive biomarkers for PD-1 inhibitor effectiveness remain lacking (15). Additionally, a paradigm shift has emerged, favoring immunotherapy followed by nonoperative management, rather than the conventional neoadjuvant chemotherapy/radiotherapy followed by surgery in patients with dMMR/MSI-H colorectal cancer (16). This evolving therapeutic approach underscores the critical importance of achieving pCR, which is essential for enabling nonoperative management. However, as there are heterogeneous responses among MSI-H/dMMR patients, there is increasing need to identify subgroups with optimal response to anti-PD-1 therapy.

Increasing data showed that obesity and overweight status may be associated with improved clinical outcomes in patients receiving immunotherapy (17, 18). However, conflicting findings exist, as some studies have reported a weak association between higher BMI and favorable treatment response (19, 20). Some studies even proposed that obesity may diminish the response rates of anti-PD-1 therapy in obese patients (21), indicating a complex interplay between adiposity and immunotherapy outcomes. Relying solely on BMI as a measure of adiposity may obscure the differential effects of various body composition components, including visceral adipose tissue, subcutaneous adipose tissue and lean muscle mass (22).

Obesity triggers a more profound and complex inflammatory response in visceral adipose tissue than in subcutaneous adipose tissue. The expansion of visceral fat leads to adipocyte hypertrophy and local hypoxia, facilitating macrophage infiltration and polarization towards a pro-inflammatory M1 phenotype. The inflamed adipose tissue can cause widespread systemic inflammation via the release of cytokines, ultimately perpetuating low-grade chronic inflammation. In essence, visceral adiposity serves as an immunomodulatory mediator, promoting inflammation and affecting immune cell function throughout the body (23, 24). The loss of skeletal muscle mass and function, known as sarcopenia, is another condition linked with immune alterations and increased circulating IL-6 and TNF-α, contributing to a state of chronic low-grade inflammation (25, 26). In addition to promoting systemic inflammation, sarcopenia disrupts the immune-regulatory functions of muscle-derived cytokines, leading to impaired T-cell activation and immune surveillance deficits (27). Myosteatosis, the infiltration of fat into skeletal muscle, integrates features of both obesity and sarcopenia and represents another crucial factor influencing inflammation and immunity (28). As a result, differences in body composition affect clinical outcomes of cancer patients across various settings; however, these effects are often not adequately captured by BMI alone (29–32).

In this study, we aim to determine whether, and through which specific body composition immunotherapy efficacy may be influenced. We utilized at-diagnosis CT imaging to comprehensively analyze body fat and lean tissue compartments in patients undergoing neoadjuvant PD-1 inhibitor therapy. By doing so, we provide one of the first reports on associations between body composition and immune checkpoint blockade (ICB) outcomes in colorectal cancer patients.

## Methods

### Patients

Consecutive patients who received preoperative PD-1 inhibitor for dMMR/MSI-H colorectal cancer between May 2019 and August 2024 were identified from Sun Yat-sen University Cancer Center. Patients were eligible for inclusion if they had histologically confirmed colorectal cancer with dMMR or MSI-H status and had anti-PD-1 therapy prior to surgical resection. Combined treatments with chemotherapy or targeted therapy were allowed. Eligible patients should also have baseline clinical and radiological data and no evidence of metastatic disease. Patients who received more than ten cycles of immune checkpoint inhibitors or did not undergo surgery were excluded from the study. This research was approved by the Institutional Review Board (IRB) of the hospital. Informed consent was waived due to the observational nature of the study.

### CT image assessment

CT images at the third lumbar vertebra (L3) level at diagnosis were analyzed using SliceOmatic software (Tomovision). Visceral adipose tissue (VAT), subcutaneous adipose tissue (SAT) and skeletal muscle were identified based on Hounsfield Unit (HU) thresholds of -150 to -50 HU, -190 to -30 HU and -29 to 150 HU, respectively. Tissue boundaries were manually corrected to ensure accurate segmentation (**Supplementary Figure 1**). Total adipose tissue (TAT) were determined as the sum of VAT and SAT, while the visceral-to-subcutaneous ratio (V/S ratio) was calculated as VAT divided by SAT. Cross-sectional areas (cm^2^) for adipose and muscle tissues were normalized to patient stature and expressed as cm^2^/m^2^, as in previous studies (33–35). Sarcopenia was defined using the cutoff point for skeletal muscle index (cm2/m2) at L3: ≤52.4 cm²/m² for men and ≤38.5 cm²/m² for women (36).

### Pathological assessment

Pathological response was assessed and quantified with tumor regression grade (TRG), which categorizes into four distinct levels: TRG0, no viable cancer cells. TRG1, presence of single cells or rare small groups of cancer cell. TRG2, Residual cancer with evident tumor regression. TRG3, extensive residual cancer with no evident tumor regression. The primary clinical outcome was treatment response, which was categorized into two groups: pCR (TRG0), indicating a complete pathological response with no viable cancer cells remaining, and non-pCR (TRG1-3), representing varying degrees of residual cancer cells.

### Statistical analysis

All statistical analyses were performed using R software. Data are presented as mean ± standard deviation (SD) or median (interquartile range). Differences of continuous variables were compared with Student’s t test. Logistic regression was analyzed using univariate and multivariate model. Body composition and other clinical variables with a p-value < 0.2 were entered into a backward conditional multivariate model and variables significantly associated with treatment response were entered into the final multivariate model. The correlation between lipid profiles and body composition variables was examined using Spearman’s correlation coefficients. The statistical significance level was set at a p-value of <0.05.

## Results

### Patient characteristics

The study included 84 patients who received PD-1 inhibitor as neoadjuvant therapy followed by surgery with a curative intent. Among these patients, 40% were female and 60% were male. The median age was 52 years. The median BMI was 22.5 kg/m^2^. All patients received at least two cycles of PD-1 inhibitors and 36.9% of the patients received ≥ 5 cycles. In addition to anti-PD-1 therapy, combination treatments with chemotherapy or targeted therapy were administered to 34 patients (40.5%). There were 77 patients who had colon cancer and 7 had rectal cancer (**Table 1**).

**Table 1:** Patient characteristics.

Variables	N=84
Age (y)	52 (39-60)
≥ 65y	12 (14.3%)
Sex	
Male	50 (59.5%)
Female	34 (40.5%)
BMI (kg/m^2^)	22.5 (20.5-24.2)
≥ 24 kg/m^2^	29 (34.5%)
Tumor location	
Ascending colon	34 (39.5%)
Transverse colon	17 (19.8%)
Descending colon	17 (19.8%)
Sigmoid colon	11 (12.8%)
Rectum	7 (8.1%)
Clinical T stage	
T2	2 (2.3%)
T3	40 (46.5%)
T4	44 (51.2%)
Therapy cycles	
≥ 5	31 (36.9%)
< 5	53 (63.1%)
Combination therapy	
Yes	34 (40.5%)
No	50 (59.5%)
Total adipose tissue (cm^2^/m^2^)	71.8 ± 37.9
Visceral adipose tissue (cm^2^/m^2^)	30.8 ± 19.5
Subcutaneous adipose tissue (cm^2^/m^2^)	40.1 ± 23.8
V/S ratio	0.85 ± 0.57
Skeletal muscle (cm^2^/m^2^)	43.9 ± 7.0
Sarcopenia	
Yes	56 (66.7%)
No	28 (33.3%)
NLR	3.4 ± 1.5
≥ 3	41 (48.8%)

Values are expressed as means ± SD, n (%) or median (interquartile range). Two patients with both an ascending colon and a sigmoid tumor are counted for once in the patient characteristics, but separately in tumor-specific analyses, resulting in a total of 84 patients and 86 tumors. TAT, total adipose tissue; VAT, visceral adipose tissue; SAT, subcutaneous adipose tissue; V/S ratio: visceral-to-subcutaneous adipose area ratio.

Coloured text highlights sarcopenia, which was defined using the cutoff point for skeletal muscle index (cm^2^/m^2^) at L3: ≤52.4 cm^2^/m^2^ for men and ≤38.5 cm^2^/m^2^ for women.

### Body composition analysis

Pathological complete response was observed in 50/86 tumors (58.1%). Univariate logistic regression identified peripheral lymphocyte count (OR = 2.20, 95% CI: 1.05-5.13, p = 0.050) and visceral adipose tissue (VAT) (OR = 1.02, 95% CI: 1.00-1.05, p = 0.047) as significant factors associated with pCR. Total adipose tissue (TAT) showed a near-significant trend (OR = 1.01, 95% CI: 0.99-1.02, p = 0.080), indicating a potential but non-significant association (**Table 2**).

**Table 2:** Association of variables with pCR in univariate and multivariate Analysis.

Variable	Univariate OR (95%CI)	p	Multivariate OR (95%CI)	p
Age (y)	1.01 (0.98-1.05)	0.60		
Sex (Male)	1.20 (0.50-2.87)	0.68		
BMI (kg/m^2^)	1.13 (0.97-1.34)	0.13		
Tumor location (Rectum)	0.96 (0.20-5.12)	0.96		
Clinical T stage (T4)	1.31(0.56-3.12)	0.54		
Combined therapy	1.57 (0.65-3.90)	0.32		
Therapy cycle (≥ 5)	1.08 (0.45-2.67)	0.86		
Neutrophil (10^9^/L)	1.01 (0.80-1.27)	0.96		
Lymphocyte (10^9^/L)	2.20 (1.05-5.13)	0.050	2.68 (1.21-6.76)	0.024
NLR	0.80 (0.59-1.06)	0.13		
TAT (cm^2^/m^2^)	1.01 (0.99-1.02)	0.080		
VAT (cm^2^/m^2^)	1.02 (1.00-1.05)	0.047	1.03 (1.01-1.06)	0.022
SAT (cm^2^/m^2^)	1.01 (0.99-1.03)	0.25		
V/S ratio	1.32 (0.61-3.18)	0.50		
Skeletal muscle (cm^2^/m^2^)	1.02 (0.96-1.08)	0.53		
Sarcopenia	1.06 (0.42-2.64)	0.90		

Clinical T stage was analyzed as T4 vs. T2–3 due to the limited number of T2 cases. TAT, total adipose tissue; VAT, visceral adipose tissue; SAT, subcutaneous adipose tissue; V/S ratio: visceral-to-subcutaneous adipose area ratio.

Other body composition variables, including BMI, total adipose tissue (TAT), subcutaneous adipose tissue (SAT), V/S ratio, skeletal muscle and sarcopenia were not significantly associated with treatment response. Additionally, tumor location (Rectum vs. Colon) and combined therapy did not impact pCR rates (**Table 2**).

**Figure 1:**
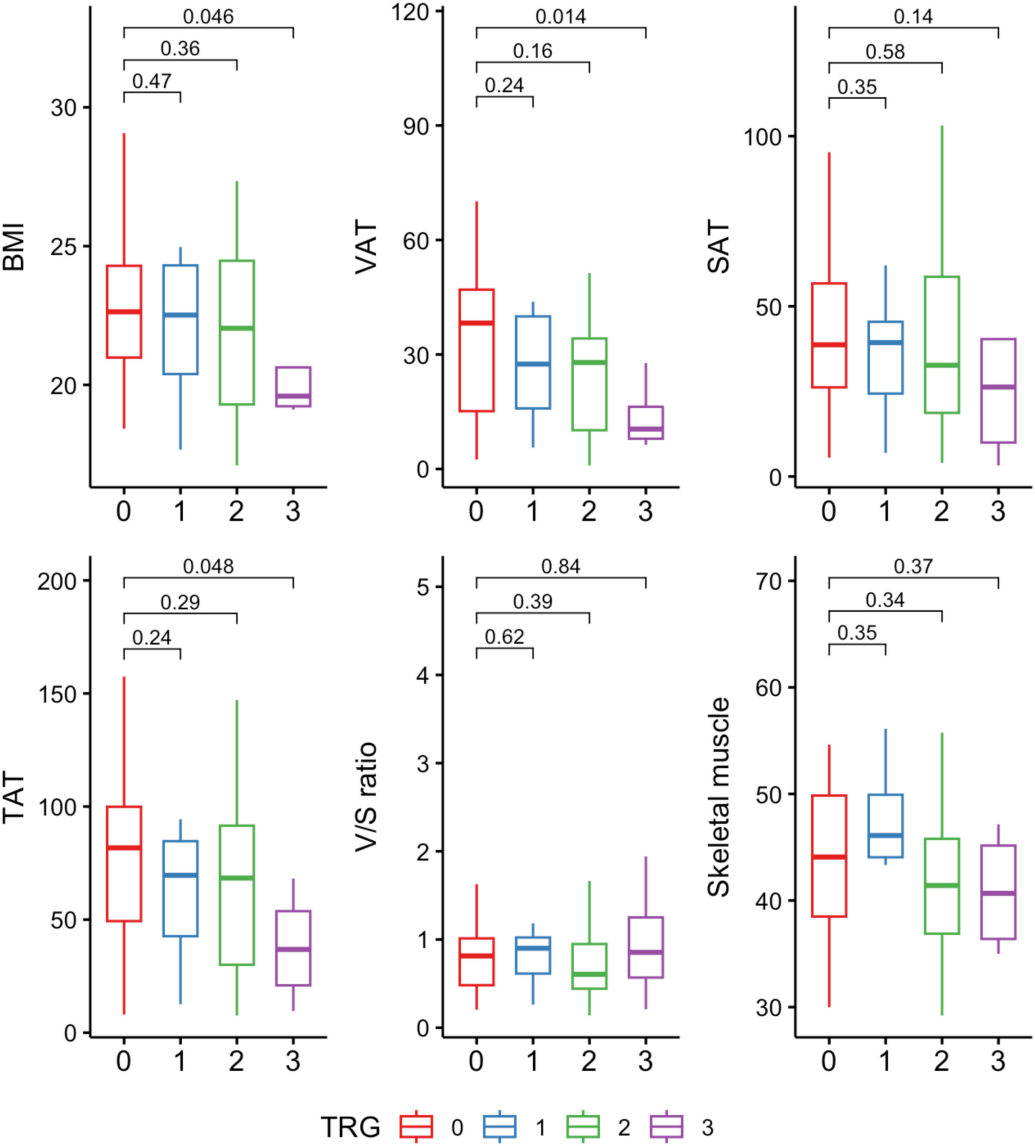
Distribution of body composition variables across tumor regression grade (TRG) categories. Units: BMI (kg/m²); VAT, SAT, TAT, skeletal muscle (cm²/m²). TAT, total adipose tissue; VAT, visceral adipose tissue; SAT, subcutaneous adipose tissue; V/S ratio, visceral-to-subcutaneous adipose tissue area ratio.

We further explored the distribution of body composition parameters across different levels of tumor regression grade (TRG). Patients with TRG0 (complete pathological response) generally exhibited higher levels of VAT compared to those with TRG1-3. Particularly, VAT showed a trend towards lower values in the TRG3 group (no tumor regression) (p=0.014), suggesting association between decreased visceral depots and non-responsive tumors. A similar trend was observed in both BMI and TAT, while subcutaneous adipose tissue (SAT), skeletal muscle, and visceral-to-subcutaneous area ratio did not show significant differences across TRG categories (**Figure 1**).

Further, multivariate logistic regression identified lymphocyte count (OR = 2.68, 95% CI: 1.21-6.76, p = 0.024) and VAT (OR = 1.03, 95% CI: 1.01-1.06, p = 0.022) as independent predictors of pCR, suggesting that immune and metabolic factors, as represented by lymphocyte counts and VAT, may play a role in determining the response to immunotherapy (**Table 2**).

### Subgroup analysis of VAT and treatment response

**Figure 2:**
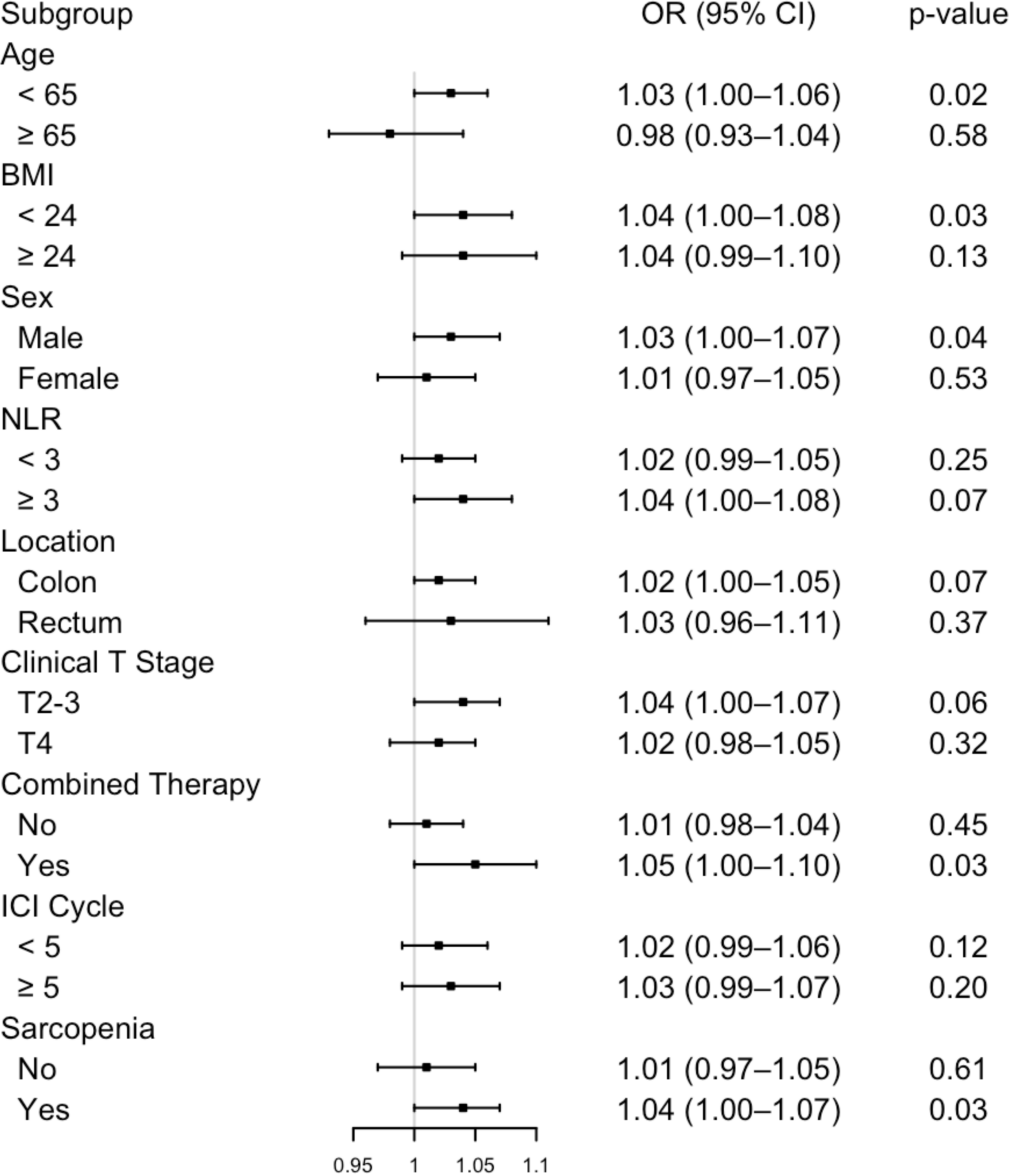
Subgroup analysis of the association between visceral adipose tissue and pathological complete response. NLR, neutrophil-to-lymphocyte ratio; ICI, immune checkpoint inhibitor.

The positive influence of visceral adipose tissue (VAT) on treatment response (pCR) was consistent across most patient subgroups. However, an opposite effect was observed in patients aged ≥ 65, indicating an age-specific variation in this association. A trend towards a stronger association between visceral adipose tissue and pathological complete response was observed in subgroups characterized by NLR ≥ 3, male, rectal cancer, clinical T2–3 stage, combined therapy, extended treatment cycles and sarcopenia (**Figure 2**).

### Correlation between body composition and lipid profiles

The strongest correlation between body composition variables and lipid profiles was observed between TG (triglycerides) and both visceral adipose tissue (VAT) (r=0.44) and total adipose tissue (TAT) (r=0.44). TG was also found to be significantly correlated with subcutaneous adipose tissue (SAT) and BMI, albeit to a lesser degree (r=0.38 and r=0.34) (**Table 3**).

**Table 3:** Correlation between body composition and lipid profiles.

Body metrics	CHO		TG		HDL		LDL		ApoA1		ApoB	
	r	p	r	p	r	p	r	p	r	p	r	p
BMI	0.05	0.63	0.34	0.002	-0.32	0.003	0.09	0.43	-0.17	0.12	0.13	0.22
TAT	0.11	0.32	0.44	<0.001	-0.21	0.05	0.11	0.31	-0.06	0.58	0.21	0.06
VAT	0.04	0.70	0.44	<0.001	-0.28	0.01	0.06	0.55	-0.12	0.27	0.14	0.19
SAT	0.15	0.17	0.38	<0.001	-0.14	0.21	0.13	0.25	0.01	0.94	0.23	0.03
V/S ratio	-0.10	0.38	0.16	0.14	-0.27	0.01	-0.04	0.74	-0.18	0.10	-0.08	0.45
Skeletal muscle	0.06	0.57	0.01	0.96	-0.13	0.22	0.10	0.35	-0.13	0.24	0.04	0.73

TAT, total adipose tissue; VAT, visceral adipose tissue; SAT, subcutaneous adipose tissue; V/S ratio: visceral-to-subcutaneous adipose area ratio; CHO, total cholesterol; TG, triglycerides; HDL, high density lipoprotein cholesterol; LDL, low density lipoprotein cholesterol; ApoA1, apolipoprotein A1; ApoB, apolipoprotein B.

## Discussion

In this study, we uncover the significant role of visceral adipose tissue (VAT) in predicting pathological complete response (pCR) in colorectal cancer patients treated with neoadjuvant immunotherapy. This is evidenced by a stepwise elevation of VAT with better tumor regression grade (TRG), suggesting a response-dependent relationship. We also identified circulating lymphocyte counts as an independent predictor of pCR, highlighting the synergistic influence of both immune and metabolic factors in augmenting the treatment efficacy of PD-1 inhibitor.

The positive association between VAT and triglycerides, along with the inverse correlation with HDL, reflects a state of metabolic dysfunction commonly seen in obesity. This pattern aligns with the “obesity paradox”, a counterintuitive phenomenon that despite being a well-established risk factor for various chronic diseases, obesity has been linked to improved outcomes in patients following diagnosis of the medical conditions, including heart failure, respiratory disease, renal disease, stroke and cancer (22, 37–40).

Once regarded as a passive energy depot, adipose tissue is now recognized as a metabolically active organ and a key regulator of endocrine signaling, interorgan communication and systemic metabolism (41). Chronic inflammation within the adipose tissue is a hallmark of obesity, along with infiltration of adipose tissue with macrophages and leukocytes (42). The abdominal adipose tissue is typically divided into the visceral and subcutaneous adipose compartments, each conferring different metabolic functions. Compared with SAT, VAT is more metabolically active, generating a different pattern of cytokines, and represents a risk factor for the development of cardiovascular disease (CVD) and type 2 diabetes (43, 44).

Visceral obesity is linked to reduction of adiponectin—an adipose cytokine with insulin-sensitizing, anti-inflammatory, and anti-fibrotic effects (41, 43, 45, 46). Our results are consistent with the notion that VAT-related adiponectin deficiency is linked to enhanced tumor-killing immunity. Experiments found that adiponectin inhibited CD8+ T cell migration *in vitro* as well as suppressed the production of IFN-γ and TNF-α. And adiponectin deficiency enhanced CD8+ T cell activation and cytotoxicity, thereby effectively restraining tumor growth (47). Moreover, adiponectin was found to modulate dendritic cells (DCs) by enhancing immunosuppressive signaling, promoting regulatory T cell (Treg) expansion, and inhibiting antigen-specific T cell responses, resulting in tumor immune escape (48, 49). Leptin, conversely, is pro-inflammatory and typically elevated in obesity. Leptin promotes T-cell proliferation and activation, and inhibits the expansion of Tregs (50, 51). In a mouse CRC model, combining exogenous leptin with anti-PD-1 further boosted tumor control and increased M1 macrophage polarization in the tumor, suggesting that leptin can fuel a more potent immune attack on the tumor in the presence of immune checkpoint blockade (52). These data suggest that a pattern of cytokines associated with visceral adiposity may modulate the tumor microenvironment, thereby impact the effects of immunotherapy.

Melanoma, non-small cell lung cancer (NSCLC), and renal cell carcinoma (RCC) were among the first malignancies to adopt ICIs into clinical practice, and most existing body composition analyses evaluating immunotherapy outcomes have been performed in these cancers. In unresectable or metastatic melanoma treated with ICIs, most studies found sarcopenia or reduced skeletal muscle density to be associated with poorer overall survival (53–56). Findings on adipose tissue compartments have been less consistent: while some studies reported no significant association (55), others suggested that visceral or total adipose tissue was detrimental to immunotherapy outcomes (53, 54). In non-oncogene-driven metastatic NSCLC treated with PD-1/PD-L1 inhibitors, higher subcutaneous fat and skeletal muscle have been linked to better clinical outcome following PD-1/PD-L1 blockade, whereas visceral and intramuscular fat showed no consistent prognostic significance (57). However, in advanced NSCLC treated with first-line anti-PD-1 therapy, higher intermuscular adipose tissue and preserved skeletal muscle were associated with improved ORR, PFS, and OS, while subcutaneous and visceral fat showed no clear impact (58).

For metastatic RCC, subcutaneous fat has been proposed as a favorable factor for ICIs treatments in some studies (59, 60), though others identified sarcopenia, rather than adipose compartments, as the dominant prognostic marker (61). These discrepancies highlight substantial heterogeneity, not only across cancer types but also within specific tumor settings. Notably, most prior studies were conducted in the metastatic stage and often involved patients exposed to previous treatments, introducing potential confounders. And survival outcomes, frequently used as primary endpoints, can be influenced by supportive care and nutritional interventions. This suggests that the context and timing of the study are critical. The distinct setting of neoadjuvant immunotherapy in our study may explain the different prognostic role of visceral adiposity, as well as the limited impact of sarcopenia observed.

In the subgroup analysis, we found that VAT-mediated immunomodulation may be amplified in the presence of more intensive treatments (combined therapy, extended treatment cycles), heightened inflammatory state (NLR ≥ 3), and earlier stage tumors (clinical T2-3), which highlights the role of VAT as a mediator in metabolic activity, systemic inflammation, and anti-tumor regimens, ultimately shaping immunotherapy outcomes. On the other hand, the reversed effect observed in patients aged ≥ 65 may be attributed to age-related immune dysfunction, referred to as immunosenescence. Older patients exhibit decline in T cell responses, impaired antigen presentation and lower sensitivity to inflammatory cytokines (62), which may weaken the ability of VAT-associated inflammatory signals to enhance anti-tumor immunity. Additionally, aging-related changes in VAT composition, including increased fibrosis, reduced metabolic activity, and altered cytokine profiles, could further affect its immunomodulatory effects (63). Thus, our study further suggests the need to account for aging in patient stratification for future investigations into immunotherapy response. Nevertheless, our observation of an age-dependent reversal in patients aged ≥ 65 was based on a limited sample size (n=12). Therefore, this intriguing trend warrants cautious interpretation and requires validation in larger cohorts.

In the overall cohort, sarcopenia was not significantly associated with treatment response in our study. However, subgroup analysis showed that the positive impact of visceral adiposity on treatment response was more pronounced in sarcopenic patients. As sarcopenia has been shown to impair immune function by promoting immune senescence (64), this finding raises the possibility that visceral adiposity may serve as an alternative metabolic reservoir to fuel antitumor immunity in the context of sarcopenia. Actionable strategies to manipulate or counterbalance this deficiency may include, in principle, a protein-rich diet to stimulate muscle protein synthesis and sufficient energy intake to replenish metabolic reserves, thereby supporting immune competence during immunotherapy. In addition, adherence to Mediterranean diets and high fiber consumption have been associated with improved immunotherapy outcomes, potentially by modulating the gut microbiome and enhancing anti-tumor immunity (65, 66). Whether these approaches yield similar benefits in colorectal cancer remains to be determined and warrants further investigation.

Our study is strengthened by the inclusion of treatment-naïve patients undergoing neoadjuvant immunotherapy, which minimizes confounding factors such as prior treatments, metabolic alterations and nutritional imbalances, allowing for a more direct observation of the association between body composition and immune checkpoint blockade response. The study also has several limitations. First, due to its retrospective nature, inherent selection bias may exist. Second, the limited sample size and the single-center, geographically homogeneous patient population may restrict the statistical power and the generalizability of the results. Third, potential confounding factors known to influence ICI efficacy, including the use of corticosteroids, antibiotics, paracetamol, celecoxib, and the composition of gut microbiome, were not incorporated into the analysis in this study (13, 67–70). Future studies with comprehensive data and broader patient diversity are needed to confirm and extend findings in our study.

## Conclusion

Higher visceral adipose tissue volume is associated with improved pathological complete response in dMMR/MSI-H colorectal cancer patients treated with PD-1 inhibitors. However, this favorable effect of visceral adiposity appears to be diminished or reversed in elderly patients (≥ 65 y), highlighting the potential influence of aging on the metabolic-immune interplay in immunotherapy response.

## Data Availability

The raw data supporting the conclusions of this article will be made available by the authors, without undue reservation.

## Supplementary material

Supplementary Figure 1 CT-based body composition analysis at the third lumbar vertebra (L3) level. Visceral adipose tissue (yellow), subcutaneous adipose tissue (blue), and skeletal muscle (red) were identified based on predefined Hounsfield unit (HU) thresholds.
