# Supplementary material for: Impact of body composition on pathological response to neoadjuvant immunotherapy in dMMR/MSI-H colorectal cancer
Source: Front Immunol. 2025 May 30;16:1589869. doi: 10.3389/fimmu.2025.1589869 (PMC12162661; doi:10.3389/fimmu.2025.1589869)
Supplement: Supplementary Figure 1 — CT-based body composition analysis at the third lumbar vertebra (L3) level. Visceral adipose tissue (yellow), subcutaneous adipose tissue (blue), and skeletal muscle (red) were identified based on predefined Hounsfield unit (HU) thresholds. [file SupplementaryFile1.docx]

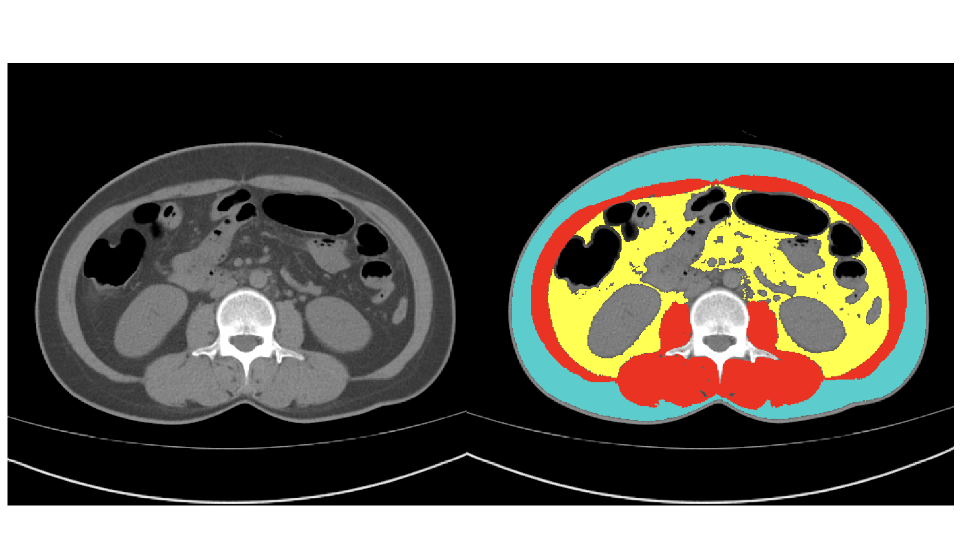


Supplementary figure 1. CT-based body composition analysis at the third lumbar vertebra (L3) level. Visceral adipose tissue (yellow), subcutaneous adipose tissue (blue), and skeletal muscle (red) were identified based on predefined Hounsfield unit (HU) thresholds.
